# Analysis of Age, Race, Ethnicity, and Sex of Participants in Clinical Trials Focused on Eating Disorders

**DOI:** 10.1001/jamanetworkopen.2022.0051

**Published:** 2022-02-21

**Authors:** Laura E. Flores, Roshell Muir, Imani Weeks, Helen Burton Murray, Julie K. Silver

**Affiliations:** 1 College of Allied Health Professions, University of Nebraska Medical Center, Omaha; 2 Division of Infectious Diseases and HIV Medicine, Department of Medicine, Drexel University College of Medicine, Philadelphia, Pennsylvania; 3 Center for Neurointestinal Health, Massachusetts General Hospital, Boston; 4 Department of Physical Medicine and Rehabilitation, Harvard Medical School, Massachusetts

## Abstract

This cross-sectional study examines the sex, racial and ethnic background, and age of participants in clinical trials focused on eating disorders conducted in the United States.

## Introduction

Studies examining the participation of women, minority racial and ethnic groups, and older individuals in clinical trials have demonstrated gaps in inclusion^1^; however, to our knowledge, this has not been studied in trials focused on eating disorders (EDs). There are known disparities in ED diagnosis, access to care, and lifetime risk.^2^ For example, people identifying as Hispanic are less likely to receive treatment than individuals from non-Hispanic groups, despite similar prevalence rates.^2^ Beyond access, current evidence-based treatments may not be appropriate for all individuals, and sociodemographic factors associated with outcomes have largely only been evaluated for adults with binge eating disorder (BED).^3^

Studying diverse populations may affect diagnosis, recommended treatments, and health outcomes. In this study, we examined the sex, racial and ethnic background, and age of participants in ED clinical trials.

## Methods

In this cross-sectional study, we collected age, sex, race, ethnicity, and primary diagnosis data from ClinicalTrials.gov for completed interventional studies with results (including children and/or adults) from January 1, 2011, to January 1, 2021, using the term *eating disorder*. The search was conducted on September 5, 2021. We excluded studies conducted outside the United States. Race and ethnicity categories were prespecified by ClinicalTrials.gov.^1^ Primary diagnoses were classified as anorexia nervosa (AN), bulimia nervosa (BN), BED, and other. Per the Common Rule, this study did not require institutional review board approval, as it was based on publicly available information. We followed Strengthening the Reporting of Observational Studies in Epidemiology (STROBE) reporting guideline for cross-sectional studies and used SPSS statistical software version 26 (IBM Corp) for descriptive analyses.

## Results

A total of 21 trials met inclusion criteria: 12 (57%) for BED, 7 (33%) for AN, 1 (5%) for BN, and 1 (5%) for other. There were 16 (76%) drug, 4 (19%) behavioral, and 1 (5%) other interventions. All studies reported age, and most included adults only (16 [76%]), with 5 (24%) including both adults and children and none including children only (Table). Three of 4 trials that allowed older adults (ie, ≥65 years) did not enroll any. Five studies (24%) included female participants only, and the remaining trials overrepresented them (3361 of 4016 total participants [83%] vs 52.1% of US population) (Figure).^4^ Thirteen trials (62%) reported race, and 9 (43%) reported ethnicity. Race and ethnicity representation varied by category (Table). Of the 1828 participants with identified race, 52 (3%) were Asian, lower than the proportion of Asian US residents (6%). Likewise, of 1629 participants with identified ethnicity, 213 (13%) were Hispanic, lower than the proportion of Hispanic US residents (19%).

**Table.** Characteristics of Registered Eating Disorder Clinical Trials in ClinicalTrials.gov and Their Participants

Characteristic	Trials, No. (%)
Total, No.	21
Intervention	
Drug	16 (76.2)
Behavioral	4 (19.0)
Other	1 (4.8)
Primary diagnosis^a^	
AN	7 (33.3)
BED	12 (57.1)
BN	1 (4.8)
Mixed population (AN and BN)^b^	1 (4.8)
Frequency of demographics reported	
Age	21 (100)
Adults only	16 (76.2)
Child or adolescent only	0
Both	5 (23.8)
Sex	21 (100)
Female only	5 (23.8)
Race	13 (61.9)
Ethnicity	9 (42.9)
**Characteristic**	**Participants, No. (%) [% of US population]^c^**
Participants in 21 trials reporting sex, No.	4016
Female,	
All trials	3361 (83.7) [50.8]
All female trials excluded	3100 (82.6) [50.8]
Participants in 13 trials reporting race, No.	1828
American Indian or Alaska Native	8 (0.4) [1.3]
Asian	52 (2.8) [5.9]
Black or African American	260 (14.2) [13.4]
Native Hawaiian or Pacific Islander	0 [0.2]
White	1435 (78.5) [76.3]
More than 1 race	45 (2.5) [2.8]
Unknown or not reported	28 (1.5) [NA]
Participants in 9 trials reporting ethnicity, No.	1629
Hispanic/Latino	213 (13.1) [18.5]

Abbreviations: AN, anorexia nervosa; BED, binge-eating disorder; BN, bulimia nervosa; NA, not applicable.

^a^ AN is characterized by restriction of energy intake associated with low body weight status, typically with fear of weight gain and body image disturbance. BN is characterized by recurrent binge eating (ie, eating a large quantity of food while feeling out of control in a discrete time period) with compensatory behaviors (eg, laxative abuse, exercise) and body image disturbance. BED is characterized by recurrent binge eating without compensatory behaviors, with significant distress. The fifth edition of the *Diagnostic and Statistical Manual of Mental Disorders* also categorizes other disorders, including avoidant/restrictive food intake disorder and presentations within the other specified feeding and eating disorders.

^b^ Mixed sample, did not report demographics separately by AN and BN groups.

^c^ Percentages from 2018 American Community Survey 1-year estimates.

**Figure.**
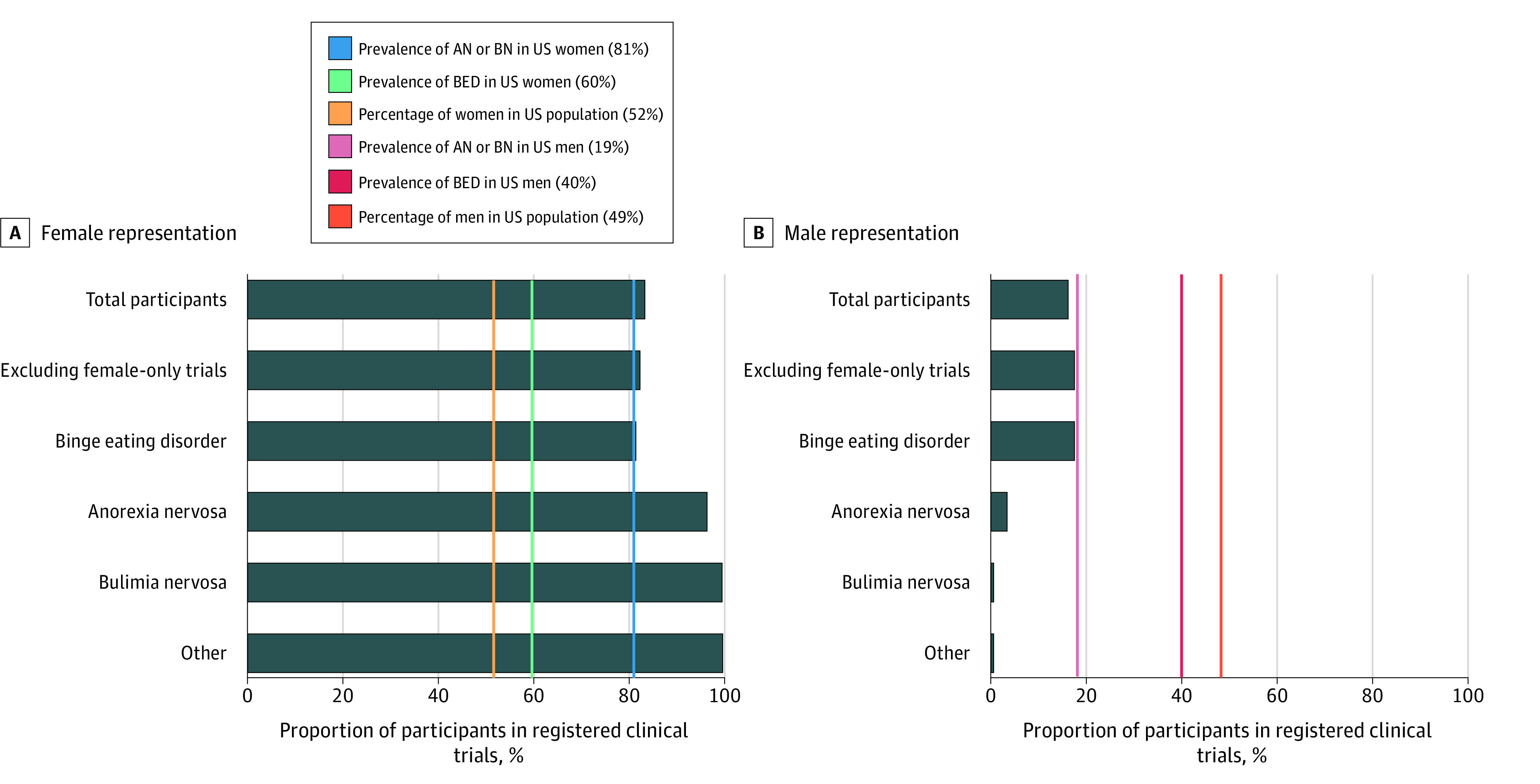
Sex Representation Among Eating Disorder Clinical Trials by Diagnostic Group Compared With Prevalence Data This figure illustrates the representation of female and male participants in registered eating disorder clinical trials compared with disease-specific prevalence rates. According to the 2018 Census, women accounted for 51.8% of the US population and men, 49.2%. Data on prevalence rates from Hudson et al.^4^

## Discussion

In this analysis of US-based clinical trials for ED during a 10-year period, we found that older adults were not participants, none of the trials focused exclusively on children and adolescents, men were underrepresented, and some racial and ethnic minority groups were underrepresented.

There is awareness of ED among young^5^ and older populations.^2^ However, our findings suggest underrepresentation of these groups in clinical trials for EDs, even though there is federal guidance to increase participation of individuals of all ages.^6^

We found that men were underrepresented overall and among each diagnostic group (Figure), despite prevalence rates of 19% in AN, 19% in BN, and 40% in BED among men.^4^ Although EDs occur more frequently in women, it is important to investigate the causes, treatments, and outcomes across the gender spectrum, including in men.^2,5^

Consistent with other studies,^1^ some racial groups were underrepresented, notably Asian, Native Hawaiian or Pacific Islander, and American Indian or Alaska Native. Regarding ethnicity, Hispanic/Latino participants were also underrepresented. Our study further supports the need to increase diverse participation in clinical trials overall and specifically in ED studies.

This study was limited to trials registered in ClinicalTrials.gov. Information about participants remains incomplete for some records. However, registries are a good source to understand characteristics of prior trials addressing ED treatments. Racial categories on the registry do not allow for disaggregation of groups, which limits information about racial subgroups.

This study suggests that populations affected by ED are not equitably reflected in clinical trials, particularly children, older adults, men, and some racial and ethnic minority groups. As ED prevention, diagnosis, and treatment may vary among different populations, it is important to address gaps in inclusion.
