# Error in Figure Key

**DOI:** 10.1001/jamanetworkopen.2022.6303

**Published:** 2022-03-14

In the Research Letter titled “Analysis of Age, Race, Ethnicity, and Sex of Participants in Clinical Trials Focused on Eating Disorders,”^1^ published February 21, 2022, there were errors in the key of the Figure. The blue, green, pink, and red lines indicate the proportion of men and women with the eating disorders listed. This article has been corrected.^1^
